# Nanomaterial-Based Drug Delivery System Targeting Lymph Nodes

**DOI:** 10.3390/pharmaceutics14071372

**Published:** 2022-06-28

**Authors:** Zesheng Cheng, Haiying Que, Li Chen, Qiu Sun, Xiawei Wei

**Affiliations:** Laboratory of Aging Research and Cancer Drug Target, State Key Laboratory of Biotherapy, National Clinical Research Center for Geriatricts, West China Hospital, Sichuan University, No. 17, Block 3, Southern Renmin Road, Chengdu 610041, China; 2019141660069@stu.scu.edu.cn (Z.C.); quehaiying@stu.scu.edu.cn (H.Q.); lichen5525@stu.scu.edu.cn (L.C.)

**Keywords:** lymph node, drug-delivery system, targeted therapy, immunology, cancer

## Abstract

The lymphatic system plays an indispensable role in humoral balance, lipid metabolism, and immune regulation. The lymph nodes (LNs) are known as the primary sites of tumor metastasis and the metastatic LNs largely affected the prognosis of the patiens. A well-designed lymphatic-targeted system favors disease treatment as well as vaccination efficacy. In recent years, development of nanotechnologies and emerging biomaterials have gained increasing attention in developing lymph-node-targeted drug-delivery systems. By mimicking the endogenous macromolecules or lipid conjugates, lymph-node-targeted nanocarries hold potential for disease diagnosis and tumor therapy. This review gives an introduction to the physiological functions of LNs and the roles of LNs in diseases, followed by a review of typical lymph-node-targeted nanomaterial-based drug-delivery systems (e.g., liposomes, micelles, inorganic nanomaterials, hydrogel, and nanocapsules). Future perspectives and conclusions concerned with lymph-node-targeted drug-delivery systems are also provided.

## 1. Introduction

One of the principal functions of the lymphatic system is to drain the interstitial fluid from the tissue and circulate lymphatic fluid through the thoracic catheter to prevent fluid accumulation and edema [1,2,3,4]. In addition, the lymphatic system is essential in immunology because one of its core functions is to provide a networked vascular system for the transport of immune cells throughout the body [1,5]. The lymphatic system comprises a network of blood vessels and nodes of circulating immune cells and provides a place for antigen presentation and immune activation [6]. The lymphatic system packages liquids, macromolecules (including proteins), particles (including infectious substances, such as bacteria), and small molecules from peripheral tissues into endogenous carriers (such as plasma lipoproteins, vesicles, or exosomes) into the systemic circulation. In immunology, lymphatic vessels transport antigens, antigen-presenting cells (APCs), and lymphocytes from tissues to drain LNs, and APCs deliver antigens to resident lymphocytes to regulate the immune response [7,8,9].

Lymph-node-targeted delivery is of great importance in cancer treatment because many solid tumors metastasize through the lymphatic system. LNs, like lymphatic vessels, have a smooth muscle layer for contraction [1]. The flow between LNs has higher resistance than other lymphatic vessels [1]. The duct system is filled with special channels that allow lymphocytes and small molecules to enter the LNs [10,11].

T cells are derived from primary lymphoid tissues and patrol the lymphatic system, vascular system, and secondary lymphoid organs until they encounter their cognate antigen. Additionally, T cells can enter nonlymphoid tissues to facilitate an inflammatory response or reside in these tissues as tissue-resident memory cells [5].

Research has clarified the effects of the lymphatic system in various diseases, such as lymphadenitis, lymph node tuberculosis, lymphoma, malignant tumor metastasis, leukemia, sarcoidosis, necrotizing lymphadenitis, and so on [3,4,5,9]. Although the lymphatic system is part of the pathology of various disease states, systemic administration with conventional drugs is challenging to achieve targeting [3]. The recognition of the key role of lymphatic vessels in diseases has led to increasing interest in targeted lymphatic transport to improve the effectiveness of treatment [4,12,13]. With our understanding of lymphatic function, the design of the lymphatic transport system has also made headway, including the imitation or integration of complex systems in the process of endogenous lymphatic transport [6].

Passive targeting preparation is a preparation that makes use of the particularity of the particle size and surface properties of the carrier to enrich the drug at a specific target or site in the body. Common nano-carriers injected intravenously interact with complement proteins or opsins in the system cycle and are easily captured and cleared by the reticuloendothelial system. If the surface is modified with recessive molecules, such as PEG, they have a long cycle in the system cycle. In normal tissues, the microvascular endothelial space is dense and intact, and macromolecules and particles do not easily pass through the blood vessel wall, while in solid tumor tissues, there are abundant blood vessels, wide vascular wall space, poor structural integrity, and loss of lymphatic reflux, resulting in selective high permeability and the retention of macromolecules and particles. This phenomenon is called the high permeability and retention effect of solid tumor tissue and referred to as the enhanced permeability and retention (EPR) effect. For example, the junction space of microvascular endothelial cells in human colon adenocarcinoma reaches 400 nm, while the average junction space of microvascular endothelial cells in normal tissues is less than 100 nm. Particles with an appropriate particle size can increase the distribution in tumor tissue [14,15,16]. 

Active targeting preparation uses a carrier that modifies or encapsulates the drug as a “missile” to transport the drug to the target to concentrate its efficacy. By connecting monoclonal antibodies, ligands, etc., this carrier can interact specifically with specific sites of the target site, change the natural distribution of particles in the body, and reach a specific target site; it can also modify the drug into pharmacological inert prodrugs—that is, pharmacological inert substances that can be activated at the active site, which can be activated at the specific target site [14,15,17]. 

The greatest difference between passive targeting preparation and active targeting preparation is that the vector construction does not contain specific molecular specific ligands, antibodies and so on [14]. 

Lymphatic targeting preparation is mainly aimed at lymphatic metastatic malignant tumor, the drug, or the drug-delivery system through local injection or systemic blood circulation with the help of lymphatic drainage to the lymph node focus to achieve the purpose of targeted or sustained-release administration of lymphoid lesions. The realization of lymphatic targeted drug delivery mainly depends on the physiological structure of the lymphatic system: the lymphatic capillaries are the initial part of the lymphatic vessels, starting from the tissue space with a dilated blind end. The walls of lymphatic capillaries are composed of monolayer endothelial cells with a large intercellular space, no basement membrane and peripheral cells, and fibrous filament pull, and thus that the lymphatic capillaries are dilated. Therefore, the permeability of lymphatic capillaries is high, and some macromolecular substances that do not easily penetrate capillaries find it easier to enter human lymphatic capillaries. When the drug is injected intramuscularly or subcutaneously or in the interstitial space between organs and tumors, the macromolecular substances with a relative molecular weight of more than 5000 have greater difficulty to enter the capillaries and are likely to enter the human lymphatic circulation through the lymphatic capillaries. They then reach the focus of the lymphatic system to achieve lymphatic targeting [15,18,19,20].

At present, progress has been made in the understanding of the role of lymphatic vessels in pathological changes and immunity, prompting people to recognize that lymphatic targeted delivery has the potential to change disease treatment and vaccination. Here, we briefly generalize the physiological function and structure of LNs and the role of LNs in health and diseases, particularly in tumor immunity. Furthermore, many strategies based on nano-drugs or materials involved in lymph-node-targeted delivery systems aiming at treating all kinds of diseases (mainly cancer) will be summarized. The development prospects and future challenges of targeted lymphatic therapy with nanomaterials are also introduced.

## 2. Physiological Function and Importance of LNs

**Figure 1:**
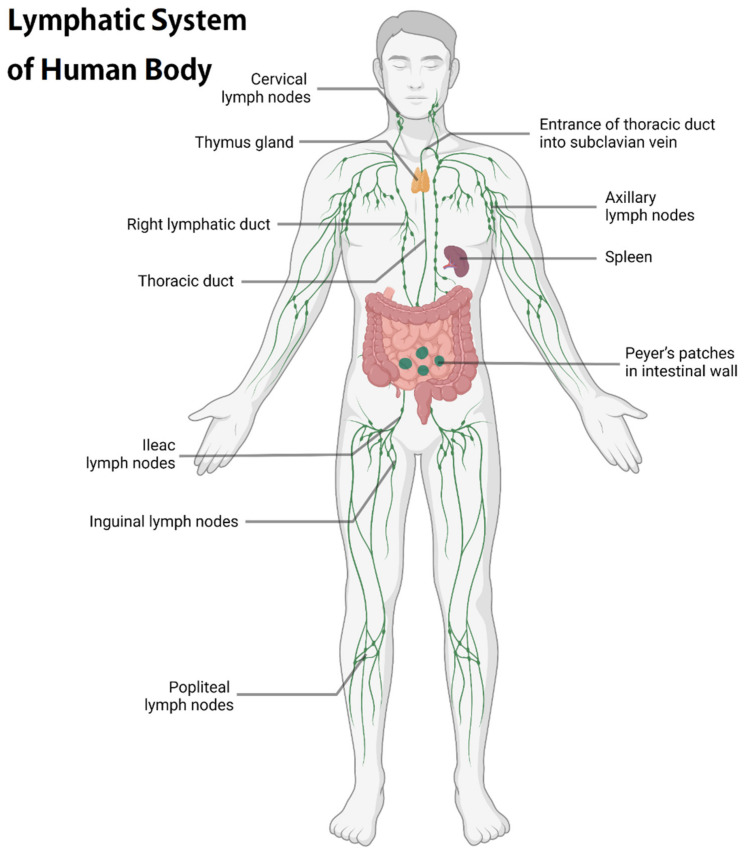
A brief illustration of the human lymphatic system.

**Figure 2:**
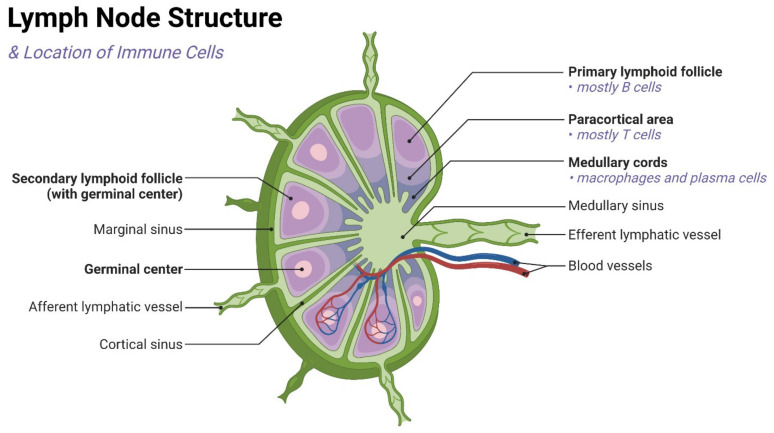
A brief schematic of lymph node anatomy and the locations of lymphocytes.

The lymphoid lobule is the basic function and anatomical unit of the LNs. The lymph node consists of multiple lymphoid lobules covering the subcapsular sinus and further wrapped in the capsule [6,21,22,23]. Figure 1 briefly shows the lymphatic system of the human body while Figure 2 briefly demonstrates the anatomical structure of the LN and the location of major immune cells in the LNs, such as dendritic cells (DCs), macrophages, T cells, and B cells. B cells exist in the follicles of the outer cortex, while T cells are situated in the paracortex, interact with APCs, and undergo clonal expansion. In adaptive immunity, the concentration of antigens, APCs, and immature lymphocytes in LNs facilitates the activation and differentiation of T cells and B cells into effector cells [21]. Most white blood cells, including effector and memory lymphocytes, activated DCs, and monocytes, can enter the afferent lymphatic vessels of the surrounding tissue [21,24,25,26,27,28,29,30,31]. Antigens and other soluble molecules in the interstitial fluid can also flow into the afferent lymphatic vessels. The molecules and cells carried by the lymph nodes are then transported to the draining lymph nodes through one-way lymphatic flow. In addition, the macrophages and sinus-associated dendritic cells of the subcapsular sinus are arranged in or under the lymphatic endothelial cells of the subcapsular sinus [28]. They are located in the lumen of the subcapsular sinus. In the cerebral cortex and accessory cerebral cortex, an interconnected network of fibroblasts and reticular cells is dispersed and is called the conduit system [29]. The countercurrent of formed lymphocytes and APCs makes it possible to quickly select antigen-specific lymphocytes from a large number of cells passing through the nodules [10]. 

Small lymph nodes contain only a few or sometimes only one lobule. The afferent lymphatic vessels are connected to the lymph nodes from one side of the afferent lymphatic vessels and are structurally divided into the cortex, accessory cortex, and medulla, and connected with the efferent lymphatic vessels. The subcapsular sinus is directly connected to the medullary sinus at the edge of the lymph node [22]. In the afferent lymphatic vessels, all sinuses and efferent lymphatic vessels are composed of a continuous layer of lymphatic endothelial cells, and the properties of lymphatic endothelial cells vary with different locations [21].

In fact, lymphatic drainage affects more than only tissue edema. For example, lymphatic drainage promotes the transport of exogenous and autoantigens to the LNs to regulate the humoral response of immunity and regulatory T cell function and immune tolerance [32], locally suppresses anti-tumor immunity [33], and guides the remodeling of draining lymph nodes [34]. The microstructure of these tissues coordinates lymphoid and lymphocyte aggregation to promote the adaptive immune response [35]. Therefore, the transport of lymphatic vessels is intrinsically related to the lymphatic function in immunophysiology [36].

Liquids, immune cells, macromolecules, and molecules are packaged as lipoproteins, vesicles, or exosomes entering the initial lymphatic vessels to form lymph. From here, the lymph flows through a network of enlarged collecting (afferent) lymphatic vessels, lymph nodes, and post-nodal (efferent) lymphatic vessels, converging on the left (or right) thoracic lymphatic vessels. The lymph is emptied from the main lymphatic vessels and goes directly into the venous system. Therapeutic drugs can target the lymphatic system through mucosal, intestinal, or parenteral pathways. The mucosal transport of particulate matter causes them to be absorbed by mucosa-associated lymphoid tissue through the epithelium. Intestinal or oral fatty drugs (usually logP > 5) cause them to be incorporated into intestinal lipoprotein collections and transported to intestinal lymphatic vessels. The parenteral or interstitial transport of macromolecules causes them to enter the lymphatic capillaries as they are too large to enter the capillaries at the drainage injection site [6,12,37].

With the increasing knowledge of the diversity of physiological functions of lymphatic regulation, researchers realized that lymphatic vessels affect a wider range of diseases than previously thought [38,39,40,41]. These diseases include lymphedema, cancer and tumor metastasis, immune and inflammatory diseases, and metabolic diseases [4,42,43]. LNs are not only the fundamental organs to start a local defense against pathogens and cancer immune response but also the place of systematic defense [44]. The most important role of LNs is to provide a unique gathering place for immune cells [45]. Migratory DCs initiate acquired immunity by presenting antigen peptides on the major histocompatibility complex (MHC) molecules of draining LNs [44,46]. As a result, LNs are the main bridge from innate immunity to acquire immunity. In many of these diseases, there are changes in lymph angiogenesis, lymphatic vessel density, dilation, contraction, and lymphatic flow, even though the functional importance of these changes is unclear [6]. 

Lymphatic spread is one of the most common modes of transmission of cancer and other diseases, one of the most critical factors related to cancer mortality, and a crucial issue of cancer management [47,48,49,50]. Tumor cells, viruses, and bacteria spread through lymphatic vessels, enter the systemic circulation, and form secondary tumors and infection sites [51,52,53]. The primary tumor usually invades the draining LNs and then grows up to be a repository for further metastasis and spread of cancer cells [54,55,56,57]. Tumor cells metastatic to the LNs can invade the blood vessels in the LNs before the downstream LNs are colonized and begin systemic metastasis [58,59,60]. That means that metastatic LNs can be invoked as a source of systemic metastasis [61,62,63]. In cancers, including breast cancer, lung cancer, and squamous cell carcinoma of the head and neck, the metastatic spread of primary tumors is the most common way to transfer tumor cells to LNs through tumor-associated lymphatic vessels [6,42,47,64,65,66,67,68,69]. 

During tumor formation and growth, high angiogenic signals can lead to random tissue remodeling and the distortion, expansion, and leakage of the tumor vascular network accompanied by lymphatic proliferation [70,71]. The lymphatic vessels near the tumor are denser than normal tissue, and the lymphatic vessels can develop in the tumor. These indicate that cancer stimulates lymphangiogenesis. Studies have shown that in breast cancer patients, the disease-free survival rate and total survival rate of patients with prominent lymphatic vessel density were significantly lower than those with low lymphatic vessel density. In the tumor microenvironment, lymphangiogenesis could be couses by numerous factors, signal molecules, and certain up-regulated enzymes [64,72]. 

Recognizing that the lymphatic system, including LNs, plays a key role in our immunity, scientists are increasingly interested in delivering immune functional molecules, such as antigens and adjuvants to LNs, to induce an effective immune response. In tumor chemotherapy, specific and selective drug delivery to target organs or target cells is the ultimate goal; thus, the progress of a new nano-drug-delivery system targeting LNs has become a research hotspot [47,64]. Therefore, LNs are attractive therapeutic targets for various unmet clinical needs, including the elimination of B- and T-cell malignant tumors, a viral pool of latent infected cells, and sentinel lymph node metastasis, improving vaccines and promoting immune tolerance [73,74,75,76,77]. Localization of LNs has been demonstrated to have enhanced efficacy in various treatment environments, including cancer and transplantation [78]. In principle, lymph node targeted drug delivery carriers can improve the delivery efficiency of LNs, thereby, reducing the total dose and reducing off-target effects and toxicity [79,80]. With the LNs gathering immune cells, antigen presentation occurs to activate systemic anti-tumor immunity, and thus it is important to deliver antigens or immune activators to activate tumor immunity. Lymphatic vessels and LNs have become therapeutic targets because they are not merely frequent sites of cancer metastasis but also vulnerable to pathogens and play a central role in regulating the acquired immune response [36]. This underlines the need for a reliable way to deliver drugs to the population of cancer cells in the lymphatic vessels. 

## 3. Interaction between Nanoparticles and Innate Immune System

Nanoparticle (NP) systems have many ideal drug delivery properties, and these can enhance the delivery of hydrophobic drugs, nucleic acids or proteins, increase their circulation time and bioavailability, reduce renal degradation and clearance, and improve the therapeutic effect [81,82]. In addition, NP allows multiple components to be delivered simultaneously at the target site in a continuous manner, thereby, enhancing therapeutic synergy [83]. NP-based preparations also have many ideal immunomodulatory characteristics because NP has the inherent ability to passively target APCs by imitating the size and shape of invading pathogens and increasing the antigen uptake, processing, and cross pressure [84]. NP can be designed to suppress or enhance the immune response and is an ideal carrier for vaccine delivery, cancer immunotherapy, or allergy therapy [85].

On the one hand, for the purpose of drug delivery, exposure to nanomaterials and their interaction with the immune system may lead to unwanted reactions due to the non-specific recognition and uptake of NP by phagocytes [81,86,87]. After administration, NP will interact with a variety of biomolecules, including proteins, sugars, and lipids present in blood, lymphoid, or interstitial fluids, which cover the surface of NP to form a so-called “protein corona” [88]. This “protein crown” consists of a variety of proteins, including signal and transport proteins, apolipoproteins, clotting factors, adhesion mediators, and complements, which can regulate NP and give it unique biological characteristics [89,90]. Inadvertently recognizing NP as a foreign body may lead to the conditioning and phagocytosis of mononuclear macrophage system [91]. Affected by the properties of particles, nano-drugs can produce immunostimulatory effects by binding to specific immune cells or through specific uptake pathways. Traditional low-toxic, non-toxic nanoscale substances may cause immunotoxicity. The unique physical and chemical properties of nano-drugs can also lead to special interactions between NPs and the immune system, and participate in catalysis, oxidation, degradation, and pyrolysis. Nano-drugs have different enhancement or inhibitory effects on the immune system, which may lead to different immunotoxicities, such as adverse immune stimulation, immunosuppression, hypersensitivity, and autoimmune diseases [92]. This can affect the clearance mechanism of NP through the kidney and liver and significantly limit the half-life of NP and the bioavailability of NP [81,88,93]. Therefore, due to the low dose, the therapeutic effect of NPs at the target site will be impaired, and toxicological events may be caused by the induction of host inflammation and immunobiological response [86,94,95].

On the contrary, in vaccine development strategies, these processes may be beneficial because these particle delivery systems can mimic the size and shape of invasive pathogens [96,97]. NP can be specifically designed to recognize and promote the continuous delivery of antigens to APC and to further regulate intracellular signaling pathways to stimulate a lasting specific immune response, thereby, improving the overall efficacy of the vaccine [98]. Vaccine delivery systems targeting lymph nodes are summarized in Table 1.

When administered in vivo, the behavior of NPs, including their recognition and interaction with cell surface and endocytosis pathways, is regulated by several factors, such as the route of administration and the physical and chemical properties of NPs, including the size, shape, surface charge, surface-area-to-volume ratio and surface chemistry or bioactivity [110,111]. This determines the overall balance between NP clearance, biodistribution, tolerance, and nanotoxicity. Therefore, understanding these factors is crucial for designing and designing NP that give priority to interacting with target cells, thus, minimizing non-specific biological distribution and the resulting side effects [89,112].

(1) Particle size. For hard spherical particles, particles between 100 and 200 nanometers in size are most likely to prolong circulation because they are large enough to avoid being ingested by the liver but small enough to avoid spleen filtration. However, the design of non-spherical and/or flexible particles can significantly prolong the circulation time of particles in the body. The same general principle governs the biological distribution of these particles: for long-circulating particles, they must be avoided from being ingested by the liver and spleen. This can be achieved by engineering deformability into particles that are >300 nm or by maintaining at least one dimension of the particles at a length scale greater than 100 nm to prevent accumulation in the liver, while still maintaining at least two dimensions at <200 nm, thus, allowing the particles to navigate to the sine of the spleen [113,114].

(2) Particle shape. In some cases, the effect of particle shape may be closely related to particle size, as described for long-circulating non-spherical particles. Particle geometry also plays a key role in the process of particle internalization. Although preliminary data proven the significant effect of particle shape, the optimal parameters of engineering nanoparticles have not been determined [115,116].

(3) Surface features. This particle property plays three important roles in the function of engineering nanoparticles. First of all, it is known that surface chemistry seriously affects the conditioning process, and the conditioning process ultimately determines the response of RES. Several methods designed to bypass immune system activation are described above. Secondly, in order to achieve cell targeting, known ligands that bind to the cell surface receptors of selected cells should be included in the design of engineering nanoparticles. Third, if organelle targeting is also needed, these ligands must be incorporated into the surface design [117,118]. We briefly summarized the specific ligands or peptides used in nanoparticles in Table 2.

(4) The release of therapeutic drugs. The realization of tailor-made activation and release is still a key obstacle in the field of engineering nanoparticles. Thus far, the main strategies include enzyme degradable, pH-sensitive, or reduced unstable materials that contribute to bond breakage between the drug and the carrier or instability when the carrier reaches the desired site of action [133,134].

In addition, due to the EPR effect, nanoparticles are expected to accumulate more in tumor tissues than in healthy tissues. The EPR effect is explained by the presence of fenestration in endothelial cells and the lack of adequate lymphatic drainage in tumors [135].

## 4. Nano-Drug Delivery Platform System Targeting Lymph Nodes

**Figure 3:**
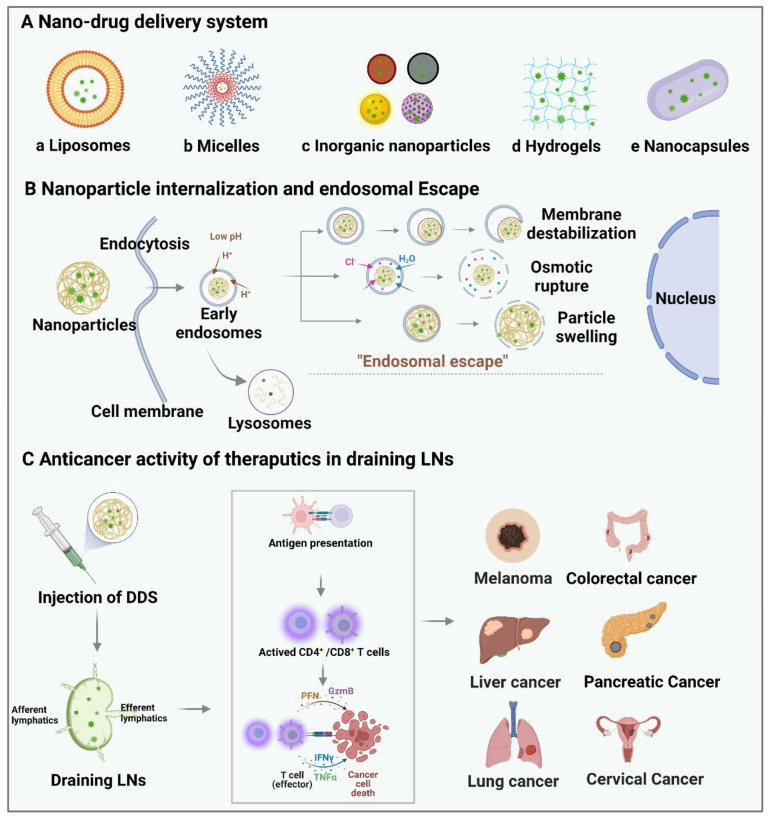
Schematic illustration of LNs targeted nano-drug-delivery system for various cancer therapy. (**A**) Typical LN-targeted nano-DDS, including liposomes, micelles, inorganic nanomaterials, hydrogel, and nanocapsules, which are loaded with therapeutics or adjuvants for targeted delivery to draining LNs. (**B**) Possible echanisms of nanoparticle endosomal escape, including membrane destabilization, osmotic rupture, and nano-cargo release with particle swelling. (**C**) After injection, DDS is efficiently drained to lymph nodes, uptake by DCs, maturating DCs, and presenting peptide-MHC I/MHC II complexes to CD8^+^/CD4^+^ T cells, respectively, activating CD4^+^ T cells and CD8^+^ T cells, thereby, eliciting robust and durable anti-tumor immunity. The targeting strategies are widely applied in the treatment of malignant tumors, such as melanoma, colorectal cancer, liver cancer, pancreatic cancer, lung cancer, and cervical cancer.

LNs are one of the most important organs for efficient antigen presentation and adaptive immune activation owing to various immune-relates cells, such as B cells, T cells, and APCs [36,136,137]. LNs also play an essential role in cell proliferation and cellular interactions. Delivering drugs to LNs shows impressive potential to interact with APCs directly, activating antibody secretion, cellular immunity, and durable anti-tumor response [138,139,140,141]. Nano-based drug-delivery systems (DDS) have been widely investigated strategies for targetability enhancement, bioavailability improvement, and prolonged circulating time [142]. Therefore, combining innovative nano-carriers with lymph-node-based therapies offers comprehensive enhancement of cancer immunotherapy and vaccination efficacy. In this part, we overview the widely used nanomaterial-based delivery systems and much recent research progress, which may be helpful for the rational design of future LN-targeted DDS (Figure 3). 

Selective delivery of therapeutic drugs to LNs may address a variety of unmet clinical needs. However, it is difficult to transport goods to specific cells in the LN cortex and paracortex due to the unique structure of the lymphatic vessels and the size-limited nature of the reticular network of the LNs. LNs are an important target of tumor vaccines. After subcutaneous or intradermal injection, 10~100 nm particles with neutral or negative surface charge are more suitable for lymphatic metastasis. However, their limited uptake by APCs and insufficient retention in LNs undoubtedly inhibit their ability to activate T-cell immunity. The benefits and limitations of different types of nanomaterial-based drug-delivery systems are demonstrated in Table 3.

### 4.1. Liposome-Related Nano-Drug Delivery Design

Liposomes, or phospholipid vesicles, were found by Bangham and his colleagues in the 1960s [143]. These vesicles are lipid NPs composed of lipid bilayers and have a hollow structure [144,145,146,147,148,149]. A variety of drugs can be wrapped in lipid bilayers or wrapped in hollow structures themselves [150,151,152,153,154]. Although liposomes have good biocompatibility, more efforts are still needed to target metastatic molecules to LNs, for instance, well-controlled size, charge, modification of polyethylene glycol (PEG), and APC-targeting ligands [23,125,155,156]. Liposomes that target LNs offer a robust approach for effective immune activation in respect of vaccine delivery and anti-tumor treatment [157]. 

Most subunit vaccines require adjuvants to promote antigen uptake and induce measurable immune responses with minimal toxicity [158]. Liposomes can be loaded with various substances and transmitted safely in vivo, which has been widely developed as vaccine adjuvants [158,159]. The liposome prepared from dimethyldioctadecylammonium bromide (DDAB) and trehalose 6-biphenyl acetate (TDB) is an effective vaccine adjuvant. Roces et al. developed a microfluidic method to produce cationic liposome adjuvants. Compared with cationic liposome adjuvants produced by small-scale lipid hydration, this method has similar biological distribution and immunogenicity. It is reported that small unilamellar liposomes composed of dimethyldioctadecylammonium (DDA) and TDB could induce a robust CD8^+^ T-cell response. DDA:TDB liposomes can be utilized as protein vaccine adjuvants without the need for toll-like receptor (TLR) agonists, thus, avoiding the potential safety risks resulting from the clinical use of TLR agonists [159]. As a potent vaccine adjuvant, the DDAB: TDB system is also in further development. Particle size controllable and scale-independent DDAB: TDB liposomal adjuvants can be produced quickly on a microfluidic platform. Further retention time of DDAB: TDB liposomes in the draining LNs could be realized by exploiting a biotin–avidin complexation system [158,160,161,162,163]. Liu’s team developed a new antigen nano-vaccine based on polyethylene glycol phospholipid derivatives and new peptides that has strong tumor specificity and immunogenicity. This nano-vaccine strategy targeting LNs can transfer new antigens to DC and activate the tumor-specific T-cell immune response more effectively. The anti-tumor effects and safety of the new antigen nano-vaccine were verified in a melanoma mouse model, indicating that it has great potential for clinical translation. In addition, the combination of new antigen nano-vaccine with anti-PD1 antibody or Treg inhibitory peptide P60 can further enhance the effect of tumor inhibition, which provides a feasible combined strategy for tumor immunotherapy [123].

In addition to being used as adjuvants for protein vaccines, liposomes can also act as adjuvants for nucleic acid vaccines to prevent them from enzymatic degradation and promote their entry into the cytoplasm. A large number of studies have demonstrated that the liposome nucleic acid vaccine has a strong anti-tumor effect. For instance, Maeta’s group developed liposome NPs as DNA vaccines in vivo. These liposomes consist of a lipoid substance (SsPalm) that can be activated by pH change. It gathers in the draining LNs and is absorbed by DCs and macrophages, particularly medullary macrophages. It has higher gene expression activity and can induce strong anti-tumor or antiprotozoal effect and can be successfully used as a DNA vaccine for tumor and protozoa infection [164]. In another case, Warashina et al. developed a cationic lipid called YSK12-C4 and loaded it into NPs containing siRNA (YSK12-C4 multifunctional coated nano-device [YSK12-MEND]) to synthesize an efficient non-viral vector that can effectively transfer siRNA to DCs, significantly promote gene silencing in mouse DCs, and enhance tumor immunotherapy by regulating the expression of immunosuppressive genes [165]. Apart from that, cyclic dinucleotides are agonists of interferon gene stimulators and can be used as vaccine adjuvants. Melissa C. Hanson et al. encapsulated cyclic dinucleotides in PEG lipid NPs and then redirected the adjuvant to draining LNs safely, enhancing the efficacy of the adjuvant significantly. Additionally, it also enhanced the CD8^+^ T-cell response induced by polypeptide vaccine and improved the anti-tumor immunotherapy [166].

Other studies based on modified liposomes have shown unique characteristics in diagnosing and treating LN-targeted diseases. Akita’s team discovered a new granular composition containing 1-dioleoyl-n-glycerophosphate serine (PS), which can be efficiently delivered to sentinel LNs. Liposomes containing PS can effectively accumulate and retain in sentinel LNs after binding with HAase. This is a promising probe for the selective detection of sentinel LNs. PS-containing liposomes are internalized into CD169-positive macrophages, which may contribute to LN aggregation. In addition, PS liposomes for sentinel LN imaging are superior to indocyanine green, a currently available imaging agent. Since the accumulation of macrophages is the driving force for the extensive accumulation of lymph nodes, the particles may be suitable for antigen or adjuvant delivery in tumor immunotherapy [167]. Mannose and other engineering site-specific ligands showed more lymphatic localization on anti-HIV drug liposomes [3,119]. For example, Kaur et al. studied the use of surface-modified liposomes to increase the absorption of zidovudine in the lymphatic system, which is used to treat HIV. Mannose was added as the targeting part to increase the uptake of macrophages to LNs and spleen. Their results concluded that mannose-encapsulated liposomes had the largest lymphatic absorption compared with ordinary liposomes and free drugs [119]. In addition, it is worth noting that mannose is also used to target brain cancer, not only as a specific molecule of LNs.

Many research groups are devoted to improve and optimize the liposome drug-delivery system for a safer, faster, and more durable drug delivery effect. Our lab developed a LN-targeted liposome delivery system for a safe and durable anti-tumor immunity response. By conjugating cholesterol to 1V209, a small-molecule TLR7 agonist, liposomes demonstrated improved transportation ability and safety in LNs compared to 1V209 [168]. From the aspect of drug administration, Oussoren et al. evaluated the entry of subcutaneous liposomes into lymphatic vessels according to the size, lipids used, and dose. When liposomes smaller than 150 nm enter the lymphatic vessels, the lymphatic absorption of neutral liposomes is limited, and the increase of dose does not affect lymphatic absorption. Oussoren’s study showed passive absorption of lymphatic vessels [169].

Liposome preparation is another breakthrough in research. Khadke et al. studied a series of liposome preparations to develop liposome lymphatic targeting systems. The results showed that the fastest clearance rate of anionic liposomes was achieved after intramuscular injection by draining lymphatic vessels. Cationic liposomes formed a reservoir at the injection site, and the monolayer vesicles possessed high lymphatic targeting. A biotin–avidin complex can promote the longer retention of liposomes in draining lymphatic vessels. In addition, microfluidic technology can be used to prepare protein liposomes, which can enhance the lymphatic targeting and retention of liposomes and embedded antigens [170]. 

### 4.2. Micellar-Based Nano Drug Delivery Platform

Micelles refer to a large number of ordered molecular aggregates of different shapes, such as spherical, layered, and rod-like micelles [171]. Micelles are made of amphiphilic monolayer molecules with both hydrophilic and hydrophobic parts and hydrophobic drugs being carried in the core of the micelle [3,149]. The hydrophobic groups of the surfactant molecules aggregate to form the core of the micelles, while the hydrophilic polar groups form the outer layer of the micelle [172]. They are usually used to deliver therapeutic molecules with poor water solubility, improve molecules’ water solubility, and prolong the blood half-life of chemotherapeutic drugs. Some antineoplastic drugs can passively accumulate at the tumor site through the leaking vascular system, thus, enhancing the permeability and retention of drugs [173,174,175].

The micelles designed in the drug-delivery system are expected to have low toxicity and suitable drug delivery mode. A polymer micelle consisting of methyl polyethylene glycol distearyl phosphatidylethanolamine (mPEG-DSPE) and Adriamycin showed an increase in Adriamycin uptake in A375 cells. The micelles injected subcutaneously are absorbed by the LNs and accumulate in the draining LNs that can kill the tumor cells in the LNs. Adriamycin can cause tissue damage; however, compared with Adriamycin alone, micelles cause less tissue damage [176]. Another kind of NP for lymphatic uptake consists of methoxy polyethylene glycol-b-polylactic acid (mPEG-PLA) and mixed poly (Dmurl-lactic acid-glycolic acid) (PLGA/mPEG-PLA). These particles transmit a small molecule called resquimod, which acts as an agonist for TLR7. Studies have demonstrated that resquimod-loaded PLGA/mPEG-PLA particles can activate anti-tumor immune response after being ingested by DCs and macrophages. If given in any other way, it has systemic toxicity; however, subcutaneous administration has no toxicity to immune cells, only cytotoxicity specific to the tumor [3,177]. 

LNs accumulate T cells and DCs, which makes them attractive for immunotherapy intervention. A large number of studies have shown that micellar-mediated molecules delivery systems targeting the LN system have favorable effects on cellular immune function [36,136,137]. In another study, Vrieze et al. designed amphiphilic lipid–polymer conjugates to deliver effective immunostimulatory small molecules to lymphoid tissues after subcutaneous administration. This can inhibit systemic inflammation and stimulate the strong immune activity of LNs, which provides a reasonable basis for the optimal design of lymphocytes targeting lipid polymer amphiphilic molecules [178]. In work done by Doddapeni, drug-loaded PEG-PCL NPs can passively target lymphatic metastasis after the proximal subcutaneous injection of a tumor [179]. Chida et al. used epirubicin micelles made of polyethylene glycol-b-polyaspartic acid (β-benzyl L-aspartic acid) to target breast cancer with axillary lymph node metastasis. Epirubicin polymer micelles pH-triggered drug release and inhibit tumor growth and axillary lymph node metastasis. The micelles are concentrated in the primary tumor and axial LNs, and epirubicin is released in the proximal part of the tumor with an acidic microenvironment [180]. The invention of new types of micelle can also achieve corresponding purposes. Zeng et al. developed hybrid particles by adjusting the physical and chemical properties of polymer hybrid micelles, which could be used to target LNs in cancer vaccines therapy. Polymer hybrid micelles are self-assembled by hydrophobic and electrostatic interactions between two amphiphilic diblock copolymers, polyethylene glycol phosphoethanolamine (PEG-PE) and polyethyleneimine-stearic acid conjugate (PSA). This overcomes the problems of limited uptake of NPs and insufficient retention of APCs in LNs, which subsequently activates T cell immunity. Zeng and colleagues successfully encapsulated melanoma antigen peptide tyrosinase-associated protein 2 (Trp2) and Toll-like receptor-9 (TLR-9) agonist CpGODN in polymer hybrid micelles with a particle size smaller than 30 nm, which can effectively target proximal LNs, where their cargo can be effectively internalized by DCs and greatly expand antigen-specific cytotoxic T lymphocytes [124].

Studies have shown that targeted delivery of protein antigens to LNs by binding to micelles can enhance the cellular immune response induced by skin administration, thereby, significantly enhance the cellular immune function of antigen-specific CD8^+^, CD4^+^ T and the memory ability of CD8^+^ T cells [33,181]. Toll-like receptor 7 agonist imiquimod (R837) was effectively loaded into mesoporous dopamine (MPDA) NPs by Wang et al. Effective DC activation and a CD8^+^ T-cell response were observed, which can be used for the combination of photothermal therapy and immunotherapy, particularly in the treatment of melanoma. They modified its surface with polyvinylpyrrolidone (PVP) to improve its lymphatic drainage ability and give it a good ability for transport and retention in the proximal LNs, thus, greatly increasing the exposure of lymphatic drugs [182].

It is reported that micelles accumulate in LNs and inhibit tumor lymphatic metastasis. In addition, the growth suppression of metastatic LN tumors is closely related to DC activation and cytotoxic CD8^+^ T-cell response [183]. Thomas et al. used 30 nm polymer NPs stabilized by PluronicF-127 to target DCs in lymphatic vessels so that accumulation could be seen in tumor draining LNs. Additionally, increased CD8^+^ T cells in LNs can slow down tumor growth and indicate a low risk of LN metastasis [184]. Additionally, by covalent coupling of small molecular Toll-like receptor 7/8 agonists with amphiphilic block copolymers, the micelles can change the pharmacokinetic characteristics of drugs and achieve effective lymphatic transport. It has the connection of π–π accumulation between the aromatic part and the amphiphilic block copolymers formed by micelles, making use of the inherent serum protein binding characteristics of lipid motifs and their tendency to accumulate in lymphoid tissues [185]. 

Small micelles (<50 nm) rather than large ones may be an effective conservative treatment to inhibit lymph node metastasis, reducing recurrence and improving survival. The selective aggregation of nano-micelles in metastatic LNs and the effect of elastic therapy also bring new implications for the non-invasive treatment of sarcoidosis. For instance, Reddy et al. used pluronic-stabilized polypropylene sulfide (PPS) NPs on the platform of antigen transfer NPs. After intradermal injection, interstitial flow efficiently delivers ultrafine NPs (25 nm) to lymphatic capillaries and draining LNs, targeting dendritic cells in half of the LNs. The surface chemistry of these NPs activates complement cascades, produces danger signals in situ, and effectively activates DCs [186,187]. In the meantime, Cabral’s group demonstrated that, in syngeneic melanoma models, sub-50 nm polymer micelles can target lymph node metastasis even after the systemic injection of platinum anticancer drugs, which limits the growth of metastasis. As the larger nano-carrier cannot penetrate the transfer site, the size of the nano-carrier is crucial for whether it can reach the metastasis site or not. This selective aggregation in metastatic LNs and its elastic therapeutic effect indicate that polymer micelles as nano-carriers have potential in the non-invasive treatment of nodular diseases. Therefore, polymer micelles smaller than 50 nm are likely to develop effective conservative treatments to prevent lymph node metastasis, reducing recurrence and improving survival [188]. Li’s team used two amphiphilic diblock copolymers, polycaprolactone-polyethyleneimine (PCL-PEI), PCL-PEG micelles loaded with Trp2 peptides and CpG oligonucleotides as adjuvants and found that they had low toxicity and high efficacy on DCs [189]. Wang et al. combined polymer micelles with tumor lymphatic homing peptide (LYP-1). LYP-1 is more targeted at tumor lymphatic vessels and gathers near blood vessels. In addition, LYP-1 micelles have the best anti-tumor effect in vitro [126]. In another study by Luo et al., LYP-1-coupled PEG-PLGA NPs were used. They compared LYP-1-coupled NPs with unbound LYP-1 NPs and found that the distribution of LYP-1 NPs in metastatic LNs was significantly higher than that of unbound NPs [127].

As mentioned earlier, micelles play an important role in the immune function of the body. Thus far, studies have shown that micelles can be used in vaccine research and production. Jewell et al. encapsulated Toll-like receptor-3 ligand poly (inosinic acid: cytidine) (PolyIC) in biodegradable poly (lactide-glycolide) particles that maintained an extracellular state and were released in LNs for several days, which could prolong the residence time of PolyIC in LNs, lead to the accumulation of Toll-like receptor agonists in lymph node resident APCs, and activate DCs more persistently. Therefore, the micelle system will produce a certain immune enhancement effect, which can be used as a widely applicable strategy to enhance therapeutic or prophylactic vaccines [120]. In another study, Jeanbart et al. used NPs that bind to tumor-associated antigens or CpG as vaccines. Compared with the non-targeted vaccine, the vaccine targeting tumor draining LNs locally and systematically increased the cytotoxic CD8^+^ T-cell response [190].

### 4.3. Inorganic Nanoparticles-Based Delivery Systems

Inorganic materials, such as gold, iron, and silica have been used to construct nanostructured materials for a variety of drug delivery and imaging. These inorganic NPs are formulated accurately and featured in different sizes, structures, and geometric shapes. On the basis of the matrix material itself, inorganic NPs have unique properties, including physical, electrical, magnetic, and optical properties [191,192,193]. Inorganic nano-carriers deliver therapeutic molecules to specific tumor sites, mainly relying on afferent lymphatic vessels. Primary and metastatic tumors destroy the normal structure of lymph nodes, resulting in increased fluid and molecular diffusion, allowing drug carriers to penetrate deeper in these lymph nodes than in healthy lymph nodes. Combined with photothermotherapy (PDT), NPs accumulated in LN tumors can exert their anti-tumor effects by heat-induced drug activation, thus, reducing the side effects [138,194,195,196].

Due to the differences in dose level, route, purity, and administration frequency of published studies, it is often challenging to accurately compare the toxicity of inorganic NPs. In addition to the common properties, such as particle size, surface area, and charge, each kind of nanomaterials may have the property of toxicity through unique mechanisms. Inflammation and induced oxidative stress are some of the common mechanisms of toxicity of inorganic nanomaterials. Long-term exposure to inorganic particles can damage the clearance, inflammation, and fibrosis [197]. With regard to the application of inorganic NPs in drug delivery and biomedical applications, more emphasis has been placed on the successful application of these nanomaterials than on their toxicity. Inorganic NPs clearly have some potential in this field. Understanding the biological distribution and elimination of NPs over time helps to design systems to deliver drugs effectively in the required time and to limit the adverse effects of NPs [198].

#### 4.3.1. Gold Nanoparticles

**Figure 4:**
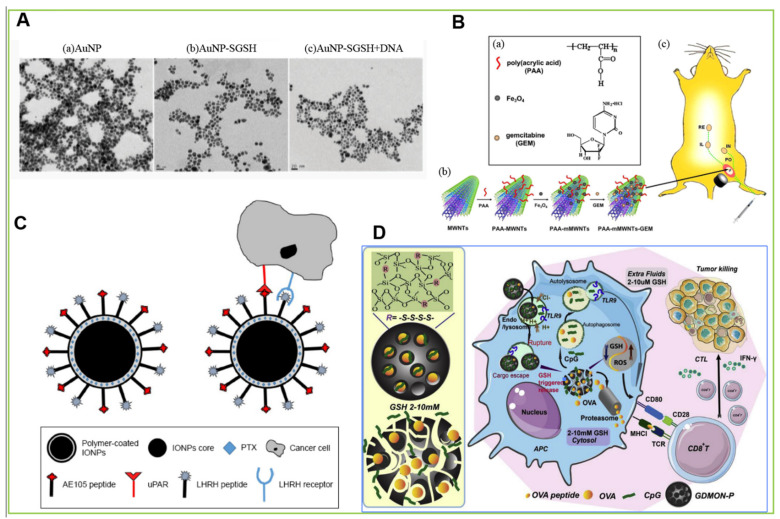
Schematic representation of functional inorganic nanoparticle-based drug-delivery systems targeting LNs. (**A**) Transmission electron microscope (TEM) images of modified AuNPs. Reproduced from Suresh Kumar Gulla et al. [122], which is licensed under the Creative Commons License. (**B**) Schematic drawings of the preparation of magnetic multiwalled carbon nanotubes (mMWNTs) and the magnetic LN-targeted chemotherapy in a murine model. Reproduced from Feng Yang et al. [225], which is licensed under Elsevier. (**C**) A brief illustration of the interaction of double-receptor-targeting IONPs conjugated with LHRH and AE105 peptides with a cancer cell. Reproduced from Md shakir Uddin ahmed et al. [128], which is licensed under the Creative Commons Attribution—Non Commercial (unported, v3.0) License. (**D**) Schematic drawings of GDMON -P+OVA+CpG enhanced cancer immunotherapy. Functionalized GDMON can transport antigenic proteins OVA and TLR9 agonists to APCs and induce endosome escape. Reproduced from Yao Lu et al. [220], which is licensed under Copyright © 2022 Elsevier B.V.

Gold nanoparticles (AuNPs) are among the most common inorganic nanomaterials, with various carrier forms, such as nanospheres, nanorods, nanoscales, nanoshells, and nanocages. Due to their excellent drug loading capacity, unique surface properties, and natural adjuvants, AuNPs have received great attention in vaccine delivery and cancer immunotherapy [122,141]. In Suresh Kumar Gulla et al. research (Figure 4A), TEM images of the gold nanoconjugates showing the morphology of the (b) positively charged bare AuNPs, (c) positively charged AuNP-SGSH nanoconjugates and (d) AuNP-SGSH + DNA nanoplex.

The size of AuNPs can be adjusted to optimize in vivo behavior, showing good lymphatic drainage and absorption. Oladipo’s research developed neutral polyethylene glycol polyalloy nanorods with a diameter of about 10 nm, which can be transported to tumors in LNs through lymphatic vessels, thus, achieving local photothermal therapy. Gold nanorods gathered rapidly in the LNs and remained near the axillary lymph nodes at the injection site. The combination of gold nanorods and PDT has clear inhibitory effects on the tumor metastasis of LN, which provides an alternative strategy for systemic drug administration [196]. 

Studies have shown that AuNPs can enhance the cellular immune response of the body through skin administration to achieve anti-tumor effects. Mottas’s group made use of amphiphilic AuNPs coated with octyl mercaptan and 11-mercaptoundecane sulfonic acid to transport TLR7 ligands as immune stimulants for tumor draining LNs. When injected subcutaneously, they can cause local immune activation, stimulating the response of cytotoxic T cells to tumors. Compared with free administration, the NPs treatment group inhibited the growth of large tumors and prolonged the survival time [121].

AuNPs have also been modified with various types of cell membranes, including erythrocyte membranes, tumor cell membranes, and platelet membranes. For example, Gao et al. used Escherichia coli membrane to wrap AuNPs to make an antibacterial vaccine containing about 40 nm particles. These particles were injected subcutaneously into mice and transported to draining LNs. They induced rapid activation and maturation of DCs, thereby, resulting in a strong antibody response and a response of Th1-and Th17-based cells to *E. coli*. These results may reflect the appropriate size of NPs and the inherent adjuvant ability of bacteria [199]. The success of this method increases the possibility of encapsulating vaccine delivery vectors with membranes collected from immune cells, such as DCs, macrophages, T cells, B cells, and NK cells, and offers a promising strategy in inducing or regulating the immune response [200,201,202,203]. 

In the tumor prevention and treatment model, AuNP-ovalbumin (AuNP-OVA) can induce an effective antigen-specific immune response even in the absence of CpG, which can effectively inhibit tumors and improve the survival rate [204]. Gulla et al. reported the design, synthesis, physicochemical characterization, and biological activity of gold NPs (Au-SGSH) covalently functionalized by thiol ligands containing shikimoyl and guanidine groups. Studies have shown that mannose-like shikimylgold NPs (Au-SGSH) covalently grafted with mannose receptors can effectively target DNA vaccines to APCs and could play an essential role in inducing an anti-tumor immune response in vivo. In a preventive environment, Au-SGSHpCMV-MART1 nanocomposites were used to generate a long-term immune response to melanoma in mice [122].

#### 4.3.2. Iron Oxide Nanoparticles

Among the various types of nanomaterials investigated, magnetic iron oxide nanoparticles (IONs) have been widely researched because of their inherent magnetism–superparamagnetism so that they can be utilized in all kinds of scientific fields, such as electronics or the environment [205,206,207]. Making use of the magnetism of IONs, targeted-site drug delivery can be accomplished by guiding IONs under the action of a localized external magnetic field [208]. This approach has been proven to be effective for the accumulation of NPs in specific pathological tissues, such as tumors or inflammatory sites [209]. In addition to this remarkable magnetism, the biocompatibility, stability, and ecological affinity of IONs make them an ideal platform for biomedical applications altogether [210]. By precisely shaping the structural properties of IONs, drugs loaded on the NPs can be effectively guided and selectively delivered to the target position. This is an effective way to improve the efficacy of drug therapy by combining or loading drugs on nano-iron oxide carriers by making use of the magnetic and biological characteristics of IONs. The adverse properties of most drugs, such as poor solubility, high toxicity, non-specific administration, and a short half-life, can be overcome by coupling with IONs [211].

Zaloga et al. reported the synthesis of IONs with an average diameter of about 7 nm coated by lauric acid and human serum albumin (HSA) and adsorbed antineoplastic drug mitoxantrone on the HSA shell. These nano-carriers exhibited strengthen stability and linear drug release kinetics within 72 h [212]. In addition, Ahmed’s team produced novel dual-receptor targeted magnetic NPs for the diagnosis and treatment of prostate cancer. In this study, two peptides were used as carriers to target two overexpressed cellular proteins in prostate cancer cells: luteinizing hormone-releasing hormone receptor (LHRHR) and urokinase type plasminogen activator receptor (uPAR). These peptides are connected to IONs by forming amide bonds with polymer-coated IONs. The final double-targeted nano-carrier demonstrated a small hydrodynamic diameter, negative Zeta potential, and high drug loading of paclitaxel (PTX). The results shows that double-receptor targeted NPs can triple the cytotoxicity of cancer cells and reduce the concentration of PTX required for free drugs with similar effects by ten times [128]. Reproduced from Md shakir Uddin ahmed et al., simulated diagram shown in Figure 4C of the interaction of double-receptor-targeting IONPs conjugated with LHRH and AE105 peptides with a cancer cell. Additionally, NPs composed of iron oxide cores with biocompatible coatings can be imaged by magnetic resonance imaging (MRI) [213,214]. Kjellman et al. studied the retention of ultra-small superparamagnetic iron oxide NPs (USPIO) in LNs after subcutaneous injection. They found that 15 nm particles passed through lymphatic vessels faster and gathered in sentinel LNs earlier, and more particles were aggregated [215]. 

In order to solve the difficulty of controlled drug release of magnetic nano-carriers, several research groups have developed different strategies, not only with the use of the magnetic field effect but also to regulate the pH and temperature or biological carriers to functionalize the surface of the particles. For instance, Gautier’s group reported the research progress in the PEGylated IONs loaded with doxorubicin (DOX) via different loading methods through the pre-formed DOX-Fe2^+^ complex. DOX-Fe2^+^ complexes can bind to hydroxyl groups on the surface of NPs and dissociate under acidic pH, eventually accomplishing pH-dependent drug release. It was also demonstrated that this drug-delivery system is able to facilitate the penetration of drug into target tumors and become less susceptible to multidrug resistance (MDR) than the free drug and increase therapeutic effect. When pH = 4, the drug release kinetics increased significantly, confirming the potential application of these nano-carriers [216].

Hyperthermia is another method to develop stimulus-responsive drug delivery using IONs. A number of research groups have developed magnetic nanocarriers coated with temperature-sensitive polymers that show enhanced drug release when IONs are submitted to another magnetic field. For example, the doxorubicin-loaded chitosan coated mesoporous IONs developed by Zou showed enhanced therapeutic effects under alternative current magnetic field [217]. Another work by Quinto et al. focused on the preparation of phospholipid-PEG-coated iron oxide NPs with a core size of 14 nm. While continuously releasing Adriamycin, these nano-carriers can generate enough heat to raise the temperature to 43 °C, which demonstrates their potential and efficacy in the combination of chemotherapy and hyperthermia in the treatment of cancer [218].

#### 4.3.3. Mesoporous Silica Nanoparticles

Mesoporous silica nanoparticles (MSN) have great potential in tumor vaccine, adjuvant design, and cancer treatment due to their adjustable pore structure, easy surface modification, and good biocompatibility.

In the project of Cha, they synthesized mesoporous silica NPs (XL-MSN) with large pore size and adjustable particle size, which had high biomolecule loading and could transmit tumor antigens and danger signals to DCs during drainage. At the same time, their applications as preventive cancer vaccines were studied. The results showed that the large pore size (about 25 nm) and extra surface modification of XL-MSN resulted in a significant increase in a load of antigen protein and TLR9 agonist, the enhancement of DC activation and antigen presentation ability, and the increase of pro-inflammatory cytokine secretion. In addition, XL-MSNS co-delivery antigen and the TLR9 agonist could effectively target LN drainage and thus induce antigen-specific cytotoxic T lymphocytes and inhibit tumor growth [219].

Lu et al. developed biodegradable glutathione-deficient dendritic mesoporous organosilica nanoparticles (GDMON) as a new platform for tumor immunotherapy combined with drug delivery. Functionalized GDMON can transport antigenic proteins OVA and TLR9 agonists to APCs and induce endosome escape. Given the advantages of a functional tetrasulfide bridging cytoskeleton, large pore size, an inherent helper, and degradability, these functional nanomaterials can not only be used as carriers to transfer antigens or oligonucleotides to APCs but also can change the intracellular microenvironment by inducing glutathione (GSH) depletion and ROS levels induced by-S-S-/GSH redox chemistry, thus, promoting cytotoxic T lymphocyte proliferation and inhibiting tumor growth [220]. In Figure 4D, Schematic illustration of GDMON-P þ OVA þ CpG enhanced cancer immunotherapy. GDMON-P are capable of co-delivering an antigen protein (ovalbumin) and CpG into APCs and inducing endosome escape. In the cytosol of APCs, GDMON-P diminish the intracellular GSH level through the -S-S-/GSH redox chemistry and thus increase ROS generation level, facilitating specific cytotoxic T cell proliferation and inducing tumour cell killing.

Mooney’s lab reported a simple way to enhance antigen immunogenicity by adsorbing polyethyleneimine (PEI) in mesoporous silica microrod (MSR) vaccines. Compared with the existing MSR vaccine and mass injection vaccine, the MSR-PEI vaccine significantly enhanced the activation of host dendritic cells and T-cell response. Impressively, a single injection of the MSR-PEI vaccine using E7 peptide completely eradicated the established large TC-1 tumors in about 80 per cent of mice and created immune memories. When immunized with B16F10 or the CT26 new antigen pool, the MSR-PEI vaccine eradicated established lung metastasis, controlled tumor growth, and cooperated with anti-CTLA4 therapy. Therefore, the MSR-PEI vaccine method can be used as a simple and powerful multi-antigen platform to achieve powerful personalized cancer vaccination [129].

#### 4.3.4. Carbon Nanoparticles

Carbon-based nanomaterials have large inner spaces for drug incorporation and offer active functional groups for chemicals covalent attachment. They have the potential for drug delivery and disease therapy [221,222,223]. Polyethylene glycol oxidized graphene NPs (RGO-PEG, 20–30 nm in diameter) is a highly modular and biodegradable new antigen vaccine preparation platform that can quickly and efficiently accumulate (15–20 %ID/g) in LNs and persist within 2 h (up to 72 h). Xu et al. developed a multifunctional and versatile nano-vaccine platform that can adapt to a variety of personalized new antigen peptides, efficiently transport them to highly specific LNs, and induce new antigen-specific T-cell responses. The vaccine can generate reactive oxygen species in DCs, guide antigen treatment and presentation to T cells, and render a strong T-cell response, which lasts for 30 days only after one round of vaccination [224]. Carbon nanotubes are also used in drug delivery to achieve cancer in the lymphatic system. For example, Yang et al. loaded gemcitabine into magnetic multi-walled carbon nanotubes with a diameter of 40–60 nm and compared them with magnetic activated carbon particles. The external Fe_3_O_4_ of carbon nanotubes endows the magnetic properties of carbon nanotubes so that a subcutaneous injection of nanotubes into the hindfoot pad under the action of magnetic field can reduce lymphatic metastasis. Nanotubes have stronger efficacy compared with magnetic activated carbon particles [225]. Magnetic lymphatic drug delivery system are demonstrated in Figure 4B reproduced from Feng Yang et al. (A) Molecular structures of poly(acrylic acid) and gemcitabine (GEM). (B) Schematic synthetic route of magnetic multiwalled carbon nanotubes (mMWNTs) and illustration of chemical reactions used to attach gemcitabine onto mMWNTs. (C) Schematic drawing of magnetic lymphatic targeted chemotherapy in mice. mMWNTsGEM were subcutaneously injected into a mouse that had cancer lymph node metastasis via the left rear footpad, and were taken upintolymphatic vesselsand retained inthe targetedlymphnodeunder the magnetic field. Forclarity, different partsare drawnat arbitrary scales. PO, popliteal lymph node; IN, inguinal lymph node; IL, para-iliac lymph node; RE, renal hilar lymph nodes.

#### 4.3.5. Other Inorganic Nanoparticles

The inverse microemulsion method prepared zinc phosphate NPs for specific delivery to LNs. Zhuang et al. coated zinc phosphate NPs with monophospholipids and loaded H-2kb-restricted peptides Trp2180-188 and H-2DB-restricted peptides Hgp10025-33. The use of these two peptides ensures the existence of multiple epitopes of MHC-mediated antigen display, making it more difficult for tumors to escape immune surveillance. In a subcutaneous melanoma model and lung metastatic melanoma model, 30 nm lipid-coated NPs were effectively excreted into LNs after intradermal administration, which induced CD8^+^ T-cell response and inhibited tumor growth [130]. In another work of Liu and He, they prepared zinc bisphosphonate NPs for LN-targeted cancer chemotherapy and PDT. It is possible to further develop these NPs into vaccine vectors [226,227]. In Li’s study, OVA modified α-alumina NPs could expel 60 NPs to LNs and transport them to autophagosomes of DCs. In the absence of other adjuvants, NPs induced effective autophagy-dependent cross-presentation and a strong anti-tumor response [228]. 

In addition, Gondan’s team used PolyIC and imiquimod (R837) as agonists of TLR3 and TLR7, respectively, combined with model antigen OVA and zinc-loaded ferromagnetic nano-phospholipid micelles, directly activating the immune response through TLR connection to achieve the purpose of killing cancer cells. The results showed that the combined action of TLR agonists induced a potent innate immune response in LNs, which had a good therapeutic effect on invasive B16-F10 melanoma cells expressing OVA [229].

### 4.4. Nano-Drug Delivery System Based on Hydrogels

Hydrogel is a three-dimensional network composed of cross-linked hydrophilic polymer chains [3]. Injectable biodegradable hydrogels can be formed in situ and have been widely used in biomedical applications, such as drug delivery and tissue engineering [230,231,232,233,234,235,236]. The intratumoral administration of injectable biodegradable hydrogel has attracted wide attention because of its continuous and controllable drug release at specific tumor sites. It has the advantages of minimizing systemic adverse drug reactions, reducing drug dosage, making it easier for drugs to reach the tumor site, and so on [237,238]. As long as the gel formula is correct, these are promising nanocarriers for targeting and delivering drugs to the lymphatic system [3,239].

Hydrogel therapy has better targeting specificity and drug-distribution characteristics and can also reduce drug toxicity. Muraoka et al. prepared cholesterol pullulan nanogels from synthetic long peptide antigens and injected them subcutaneously in mice. The results showed that the peptides were drained to local LNs and absorbed by macrophages in the nodular medulla. As this peptide is only specifically absorbed by macrophages located in the medulla but not by immune cells located in the stroma or other parts of the lymph nodes, the preparation presented CD8+T cell antigen and inhibited tumor growth [240]. In addition, lipid-based nanocapsules in hydrogels have brought satisfactory news in terms of lymphatic targeting of Wauthoz. In the in situ non-small-cell lung cancer model of immunodeficient mice, the subcutaneous or intravenous injection of lauryl derivatives of gemcitabine can target the lymphatic system, thereby, reducing the toxicity associated with gemcitabine treatment and inhibiting mediastinal metastasis. Their experimental results showed that subcutaneous hydrogel nanocapsules have higher specificity and controlled release properties for lymphatic vessels compared with intravenous nanocapsules [241,242]. 

Hydrogel-based drug-delivery systems can also prolong the action time of drugs in vivo and induce humoral and cellular immune responses and thus have good clinical application prospects in immunotherapy. For instance, Nuhn et al. proved that the small molecule TLR7/8 agonist based on Imidazoline was covalently linked with degradable polymer hydrogel NPs with a particle size of 50 nm, and the activity of activating TLR7/8 in DC in vitro remained basically unchanged. An imidazoline-coupled nano-gel combined the effective trigger of TLR7/8 with the immune activation concentration of the local injection site and draining lymph nodes, which induced a better antibody and T-cell response to a tuberculosis antigen, thus, greatly reducing the systemic inflammatory response. In anticancer immunotherapy, intratumoral injection of immunostimulatory nanogels may improve the therapeutic benefit of local application of imidazolines [243]. Additionally, a study by Song et al. tested a polypeptide hydrogel made from injectable polyethylene glycol poly (L-valine) for immunotherapy of melanoma. This is a three-dimensional porous hydrogel with the ability to recruit DCs. The tumor cell lysates were loaded into the hydrogel as antigens and TLR3 agonists (polyinosine:polycytidine monophosphate) and then released slowly so that the recruited DCs were activated. The hydrogel can prolong the time of antigen at the injection site and increase the number of LNs. The subcutaneous injection can induce cytotoxic T lymphocyte reaction and increase the number of CD8^+^ T cells in draining LNs, which has a good anti-melanoma effect in vivo [131]. 

In addition to being used in immunotherapy, hydrogels with high specific targeting and efficient antigen presentation ability can effectively recruit immune cells, which is of great guiding significance for the design and preparation of cancer vaccines. In research by Koker’s group, they demonstrated that polyethylene glycol can significantly improve the lymph node targeting of hydrogel NPs and reported the design of polymer hydrogel NPs that can target multiple immune cell subsets in LNs effectively. The increase of granules in LNs led to an increase in the initiation of antigen-specific T cells. They prepared NPs by permeating mesoporous silica particles (about 200 nm) with polymethacrylic acid, followed by disulfide bond cross-linking and template removal. Compared with the use of polymethacrylic acid NPs alone, because polyethylene glycol increases lymphatic drainage, PEG polymethacrylic acid NPs successfully deliver peptides and improve the ability of antigen presentation. Therefore, PEG-modified hydrogels may be helpful to deliver cancer vaccines directly to the lymphatic system [244]. Apart from that, Verbeke’s team used injectable porous hydrogels to deliver BDC peptides in type I diabetic mice. BDC peptides are released in the form of poly (dodecanolamide) (PLGA) microspheres or coupled with alginate polymers. In their experiment, AuNPs loaded with granulocyte-macrophage colony-stimulating factor (GM-CSF) and polypeptide PLGA microspheres were loaded into a pore-forming gel, and a significant increase of antigen-specific CD4^+^ T cells was detected in drained LNs. This work suggests that such a platform can be used to affect the presence of immune cells in draining LNs and may contribute to cancer vaccination [132]. 

### 4.5. New Type of High-Efficiency Drug Delivery Nanocapsules

Nanocapsules have been extensively documented as lymphatic targeting drug delivery carriers through controlling several essential factors, such as the size, distribution, biocompatibility, and stimuli-response [3,241,242,245]. 

The size of nanocapsules has a great influence on the biological distribution of drugs and the action time of drugs. Vicente’s team reported that small-size (100 nm) polyamino acid nanocapsules had better biodistribution and faster access to lymphatic vessels than did 200 nm nanocapsules. At the same time, 100 nm nanocapsules demonstrated sufficient docetaxel loading and sustained release characteristics. In addition, another kind of nanocapsules made from polysaccharide shells were slowly excreted from the injection site and accumulated in the draining LNs. The nanocapsules can form a repository at the injection site with slow lymphatic drainage and long-term lymphatic retention [246]. 

The customized nanocapsules have the characteristics of narrow particle size distribution and good biocompatibility and can easily load antigens and adjuvants. Li’s research found that lipid nanocapsules containing protein or peptide antigens promoted the uptake of APCs and the transport of APCs to draining LNs. Compared with soluble antigens and adjuvants, the combination of nanocapsules loaded with these antigens and Toll-like receptor agonists can improve the therapeutic efficacy of tumor vaccines and prophylactic virus vaccines [247]. 

In addition, nanocapsules can improve the oral bioavailability of insoluble drugs. Attili-Qadri et al. found that the oral bioavailability of docetaxel can be improved by lymphatic transport. Oral docetaxel nanocapsules are coated with apolipoproteins and phospholipids when they pass through intestinal cells and are transported to intestinal lymphatic vessels, resulting in a significant increase in the exposure time [248]. 

### 4.6. Endogenous Nanocarriers for Targeted Therapy

Exosomes refer to a class of secretory nanoparticles defined by their size, surface protein, and fat composition as well as the ability to carry RNA and protein. They are important media for cell-to-cell communication and cell niche regulators, and are now considered to be a unique cellular entity that can carry goods, such as RNA, proteins, lipids and so on to share among cells [249,250]. Their altered characteristics in many diseases, such as cancer, suggest that they are important for diagnostic and therapeutic purposes, thus, prompting researchers to use exosomes as drug delivery carriers, particularly for gene therapy. Due to the endogenous source, exosome-based drug-delivery systems may have advantages in the treatment of cancer; however, the design needs to be further improved to prove that its use at a clinical scale is reasonable [251].

Exosomes should be able to carry a large amount of treatment cargo in order to qualify as a drug delivery carrier. It has now been shown that a variety of goods show therapeutic effects after exoskeleton-based delivery to specific tissues. Most studies shown in Table 4 have taken advantage of an important physiological feature of the exosome (interfering with the transfer of RNA), while a few studies have explored the possibility of loading other types of therapeutic cargo into exosome particles.

### 4.7. Other Novel Targeted Delivery Nanoparticles

The combination of chemical reactions with nanomedicine can be a new application of nano-carriers and has a certain effect on the absorption and distribution of drugs. In a recent study, Schudel et al. developed a synthetic nano-carrier system that brings mercaptan-reactive oxyboradiene (OND) joints to nano-drug-delivery systems. These joints break in a pH-and solvent-insensitive manner through a first-order Retro–Diels–Alder mechanism. First, antigenic particles are efficiently transported to the draining LNs through lymphatic vessels. Second, OND-sulfhydryl chemistry is used to attach small molecular cargos to these particles, which are released in a programmable manner and are passively diffused when they reach LNs. The system can release its payload at different rates, help to enhance lymphatic absorption and improve lymphatic transport, and promote small and medium molecules into lymphocyte subsets that are difficult to obtain in conventional drug preparations [79].

At present, there are also composite NPs with pH regulation as the design center, which not only have good targetability but also have broad prospects in the clinical treatment of inducing RNA. Based on the study of the structure–activity relationship, Sato et al. selected a suitable combination of hydrophilic head groups and hydrophobic tails to prepare lipid NPs composed of pH-sensitive cationic lipid CL4H6 (CL4H6-LNPs). pH-sensitive cationic lipids can promote hepatocyte targeting and endosome escape, seriously affect the utilization of siRNA, and make it a key substance for the effective transmission of siRNA. Cl4H6-LNPs showed higher efficiency in endosome escape, cytoplasmic release, and RNA-induced silencing of siRNAs complex. A systematic understanding of the structure–activity relationship of lipids will greatly promote the development of complex pH-sensitive cationic lipids based on siRNA therapy [271]. 

DNA NPs targeting Langerhans cells have been proven to have good immune cell recruitment and induction of cellular immunity, and researchers are constantly updating and developing better techniques. To develop new treatments for acquired immune deficiency syndrome (AIDS), Lori’s team developed a DemaVir patch that can induce a lasting HIV-specific T-cell response in immunotherapy, thus, playing a role in the treatment of AIDS. DermaVir is chemically synthesized in NPs and consists of an HIV-1 antigen coding plasmid DNA. Epidermal Langerhans cells capture NPs and transport them to draining LNs. In the process of transport, Langerhans cells mature into DCs, which can effectively present DNA-encoded antigens to immature T cells and induce cellular immunity [272]. Recently, Toke et al. developed a DNA formulation with polymers and obtained synthetic “pathogen-like” NPs, which are preferentially targeted at Langerhans cells in epidermal culture. Langerhans cells in the epidermis pick up NPs and gather them in the nuclear region, which proves the effective nuclear DNA transport in vivo. Combining NP delivery and skin therapy is essential for effectively loading vaccines into the epidermis and activating Langerhans cells to migrate to lymph nodes [245]. 

The IHIVARNA consortium conducted the first human clinical trial using naked mRNA (IHIVARNA), which combines a dendritic cell activation strategy (TriMix:CD40LTOPCD70CATLR4RNA) with a new HIV immunogen sequence (HTI immunogen). This phase I exploratory dose increment test showed that iHIVARNA vaccination was feasible, harmless, and well tolerated. It could induce a moderate HIV-specific immune response and instantly increase the expression of caHIV-RNA and hypersensitive plasma viremia. These data support further the exploration of iHIVARNA in the ongoing phase II clinical trial [273].

## 5. Nanomaterial-Based Drug Delivery Systems Targeting T Cells

T-cell-based immunotherapy is expected to treat many types of cancer, with three approved B-cell malignant tumor products and a large number of treatment lines in clinical trials. However, their widespread implementation faces several challenges. These problems include the insufficient expansion of adoptive metastatic T cells, inefficient transport of T cells to solid tumors, decreased T-cell activity due to a poor tumor microenvironment, and the loss of target antigen expression. Together, these factors limit the number of tumor-related therapeutic active T cells. Nanomaterials are the only materials suitable for overcoming these challenges because they can be reasonably designed to enhance T-cell expansion, overcome complex physical barriers, and regulate the tumor microenvironment [274]. Studies of nanomaterial-based T-cell cancer immunotherapies are demonstrated in Table 5.

In short, nanomaterials are being widely explored to improve immunotherapy for T-cell cancer, and they have proven to be successful in expanding T cells in vivo, altering T-cell activity, and overcoming barriers to solid tumor delivery. These nanotechnologies that are expected to regulate the function of T cells may be more widely used in basic immunological research and clinical applications of cancer immunotherapy. The continuous optimization of nanomaterials may eventually expand the benefits of current T-cell-based cancer therapy and lead to the development of more advanced cancer immunotherapy.

## 6. Stimuli-Responsive Nanomaterials for Lymphatic System Drug Delivery in Tumor Therapy

A drug release system with controlled release allows targeted drug release, sustained or triggered drug release, and combined release of drug combinations. This helps to develop safer and more effective treatments by reducing side effects and improving pharmacokinetics and drug circulation half-life [284,285]. In addition, these systems can also prevent the degradation and excretion of therapeutic molecules, thereby, reducing the dose. Nanostructures can be customized to respond to different stimuli that lead to drug release [286,287]. The different characteristics of tumor cell microenvironment make it an ideal trigger for controlled drug release. External stimulation from the outside of the cell can also be used to trigger the release of bioactive molecules [288,289].

### 6.1. pH Stimuli

The acidic microenvironment produced by tumor cells can be used as a stimulus to release therapeutic molecules near the tumor. The PH response system can utilize the acidic pH of tumor microenvironment (pH 6.5) and intimal chamber (pH 4.5–5.5) [290,291]. In this regard, three strategies have been developed for the delivery of therapeutic drugs in tumor microenvironments, involving charge-shift polymers, conformational switches of DNA-based drug carriers, and acid-sensitive junctions for therapeutic drug coupling or acid-sensitive building blocks for the preparation of nanoparticles [292,293,294,295]. Hydrazine, imine acetal/ketal, o-ester, cis-aconityl, and b-thiopropionate are the most common parts for the preparation of pH-sensitive nano-carriers [296]. C-rich oligonucleotides showed linear conformation under physiological pH. However, under acidic pH, intermolecular or intramolecular C-quadruplets formed, resulting in conformational changes that can be used to release goods [297]. However, in order to deliver drugs effectively in this way, fine-tuning the pH-controlled release system is required, which is challenging. In particular, nanoparticles must be stabilized under physiological pH (about 7.4) to prevent non-specific drug release [298]. Once the nanostructure reaches the tumor microenvironment, low pH (about 6.5) stimulates physical and chemical changes in the nanostructure and promotes the release of its goods [299].

### 6.2. Redox Stimuli

In tumor cells, ROS levels are elevated due to a variety of factors, such as metabolic disorders, changes in mitochondrial electron transporters, hypoxia, inflammation, and carcinogenic signals [300]. Therefore, tumor cells have a complex network of antioxidants to protect cell macromolecules from the effects of ROS and to prevent cell death.

Recently, S. Chibh and colleagues used this method to develop a disulfide-bond-based targeting and redox response nanostructure through the synthesis of molecular self-assembly of dipeptides. These nanostructures are designed to specifically target tumor cells through folic acid coupling and transport the chemotherapeutic drug DOX. The existence of disulfide bonds makes the disintegration of nanoparticles and the release of DOX dependent on high levels of GSH in cancer cells. Compared with non-tumor cells, nanostructures are more effectively internalized into tumor cells that overexpress folic acid receptors [301]. Another strategy to take advantage of high levels of GSH in tumor cells is to bind disulfide bonds to the polymer backbone. Therefore, in the presence of GSH, the nanoparticles will disintegrate, and the packaged goods will be released [302].

### 6.3. Magnetic Responsive

Magnetic materials can be used to control the delivery of therapeutic drugs in different ways. For example, magnets can be used to guide nano-drugs to interested tissues, thus, significantly improving the selectivity. For this method, magnetic nanoparticles based on iron oxide are usually used because of their stability and biocompatibility in biomedical applications [303,304]. Interestingly, they can combine with liposomes, polymers, and porous metal nanocapsules to make them magnetic. A recent report by A.S.Garanina and colleagues explored the use of injectable and temperature-sensitive cobalt ferrite nanoparticles to treat colon and breast cancer. In this study, the temperature-dependent therapeutic effects of nanoparticles (magnetic hyperthermia) were compared and analyzed. The study of cell culture showed that the toxicity of this treatment increased with the increase of temperature. In addition, it was observed that colon cancer cells were more sensitive than breast cancer cells when heated to 43 °C. Interestingly, studies in animal models have shown that this mild increase in body temperature is effective for non-metastatic colon cancer. However, it did not work in mouse models of metastatic breast cancer. Notably, studies have shown that in a mouse model of breast cancer, a temperature higher than 47 degrees Celsius results in the complete removal of the primary tumor with 25% to 40% long-term survival rates [305].

### 6.4. Light Responsive

Light of various wavelengths can promote significant changes in the photoresponsive drug-delivery system, allowing drug release by adjusting the exposure time and light intensity [306,307,308,309]. In this regard, different strategies are used for drug release involving the use of photosensitive linkers that react to ultraviolet, green (540 nm), and red (645–675 nm) light, respectively, such as o-nitrophenyl, aminoacrylate, and thio-metal bonds [310,311]. In addition, the light source can also be used for other treatments, such as photodynamic therapy and photothermal therapy (PTT). J. Cao and his colleagues developed near-infrared light-triggered biodegradable amphiphilic chitosan block copolymer micelles that deliver both the antineoplastic drug PTX and the near-infrared dye cypate to the tumor site for combined chemotherapy and PTT. Near-infrared light promoted the dissociation of micelles, showing a high temperature response to PTT. It is worth noting that the release of PTX in tumor environment was significantly increased. Studies on breast cancer models in vitro and in vivo have shown that PTT and chemotherapy have a synergistic effect. This combination of near-infrared photosensitive therapy reduces the recurrence rate of cancer and contributes to sensitive imaging diagnosis [312].

### 6.5. Ultrasound Responsive

Ultrasound provides a unique trigger process for the release of therapeutic molecules based on mechanical and/or thermal effects, which are caused by cavitation and radiation [313]. In addition, this stimulation is non-invasive because it uses non-ionizing radiation, and the frequency can be adjusted to change the depth of penetration according to the depth of the desired tissue [314]. When low ultrasound frequency (within the KHz range) is applied, the cavitation effect dominates, and it can be used to trigger drug release, perfluorocarbon nanoemulsions are used to overcome the limitations of bubble lifetime and extravasation, and to promote cell uptake and/or drug release at the tumor site [315,316,317]. Another possibility is to use high-intensity focused ultrasound to increase the temperature, which can also promote drug release [318,319,320]. J.L. Paris and colleagues designed an ultrasonic response system based on doxorubicin-loaded MSN. The system consisted of a nano-lattice composed of a temperature-responsive polymer *p*(MEO_2_MA) and an ultrasonic responsive monomer to form a copolymer. The system allows nanoparticles to be loaded at low temperature (4 °C), and the copolymer adopts an open conformation at this temperature. Once the system reaches the physiological temperature (37 °C), the copolymer changes to a collapsed state, allowing the goods to remain in the pores [313].

## 7. Clinical Applications of Nano-Drugs

The first generation of NPs are mainly based on liposomes and polymer–drug conjugates. They can be functionalized, for example, by interacting with ligands of cell surface receptors to promote targeting of specific cells and tissues. In addition, they can also be coated with polymers to prolong the cycle half-life. In 1995, the U.S. Food and Drug Administration (FDA) approved the first liposome-based treatment of liposome-encapsulated Adriamycin (Doxil; OrthoBiotech, Horsham, PA, USA) for the treatment of HIV-associated Kaposi’s sarcoma and subsequently approved for the treatment of ovarian cancer and multiple myeloma. Polymer–drug conjugates have also been extensively studied, and several have been approved by regulators. PEG can improve protein solubility and plasma stability and reduce immunogenicity. Thus far, it is the most widely studied polymer. In 1994, polyethylene glycol-1-asparaginase (Enzon, Plantation, FL, USA) became the first NP therapy for acute lymphoblastic leukemia approved by the FDA. More nano-drugs are listed in Table 6. 

Although substantial progress has been made in lymphatic administration in recent years, relatively few drugs currently or previously on the market were designed to deliberately increase lymphatic administration in order to achieve pharmacokinetic or therapeutic effects. In fact, many parenteral or oral vaccines may enter the lymphatic system to promote the immune response. However, it seems that most designs do not take this feature into account. Similarly, several oral high lipophilic drugs, parenteral delivery biological agents (e.g., modified or unmodified proteins and antibodies), and macromolecular and nanoparticle delivery systems currently on the market or in clinical trials have properties that indicate the possibility of lymphatic transport; however, this has rarely been explored or utilized.

Thus far, most macromolecular biological products and drug-delivery systems have been developed for the treatment of cancer or inflammatory diseases. Therefore, the absorption of these systems through the lymphatic system may play an important role in their ability to eradicate cancer metastasis and reduce inflammation, although this has not been directly demonstrated in patients. The lack of clinical evidence for lymphatic targeting reflects the fact that the assessment of human lymphoid drug exposure is complex and therefore rarely attempted. In contrast, lymphatic transmission is most commonly studied in rodents, occasionally in larger animal species, such as pigs, dogs, and sheep. In the past, the quantification of human lymphatic transport required invasive surgery to intubate lymphatic vessels or collect lymphoid nodules. Recent advances in lymphography and minimally invasive techniques for intubation of lymphatic vessels in the human thoracic cavity indicate that more detailed studies on the collection of lymph nodes and/or quantification of human lymphatic transport are increasingly possible.

## 8. Perspectives and Disscussion

As we know more about the core role of lymphatic vessels in regulating diseases, such as cancer, transplant rejection, infection, inflammation, and metabolic disorders, increasing attention has been paid to the lymphatic system and lymphocytes as therapeutic targets. There is growing evidence supporting the benefits of therapeutic and protective vaccines for APCs in LNs and strengthening the ultimate immune response. This has become an effective strategy to deliver cargo into LNs by promoting interstitial nano-carriers to transfer to lymphatic vessels and then to LNs. The recent increase in our understanding has spurred renewed interest in the lymphatic system as a drug target, thus, providing further impetus for research in this field.

Looking ahead, drug delivery will continue to be driven by a more detailed understanding of lymphobiology, particularly the mechanisms of lymphatic absorption and entry as well as the role of lymphatic vessels in diseases. Advances in materials and pharmaceutical science—in particular the construction of macromolecular couplings and structures with specific lymphoid affinity—will further promote efforts to promote lymphatic targeting. The area of focus may be the growing recognition that lymphatic acquisition is not only a function of size but also a series of transport and metabolic processes. Finally, although lymphatic vessels and lymphoid tissue clearly play a central role in a range of diseases, it is also clear that this is highly interactive and that the same disease state affects lymphoid structure and function. Nevertheless, in most LN-delivery systems based on nanomaterials, T-cell targeting may lead to systemic effects, leading to crosstalk between LNs and the whole immune system. However, whether such systemic effects lead to unwanted immunologic side effects remains to be elucidated. Future efforts may be useful to address the effects of disease on lymphatic function changes on the lymphatic pathway of drugs, vaccines, and drug-delivery systems to better promote the development of powerful lymphotropic delivery carriers.

## 9. Conclusions

Lymphatic vessels have long been regarded as the “sewage system” for removing liquids, proteins, and fragments from the matrix, as well as the transport mechanism of dietary fat. To achieve a more precise and effective delivery of the cargos, several aspects should be considered, including carrier size, hydrophobicity, surface charge, and targeting properties. Table 7 briefly summarizes the characterizations, advantages, and applications of the different nano-drug-delivery systems presented in this review.

Based on the previous experience in exploring the possibility of provoking effective immune response by targeting LNs, we conducted the current review. Despite the introduction for the basic structure and function of LNs, we focused on the emerging LN-targeted nano-drug-delivery systems. The translational research of the targeted delivery system for LN might be underpinned by the basic research development of the related fields, such as the comprehensive understanding of various cell types in LNs and cell–cell crosstalk of LNs with other remote tissues. However, the lymph node might be an attractive potential target for immune therapy and vaccine development for clinical trials in the future and deserves to be further studied.

**Table 7:** Overview of the five types of nano-drug-delivery systems.

Type of Nano-Drug Delivery System	Combined Nanomaterials/Applied Targeting Molecules	Advantages	Therapeutic Agents	Application	Therapeutic Performance	Ref.
Liposome	DDAB and TDB	Lower potential safety risks	N/A	Vaccine adjuvants	Induce a robust CD8^+^ T-cell response	[159]
	PEG phospholipid derivatives and new peptides	Activate tumor-specific T-cell immune response more effectively	Anti-PD1 antibody or Treg inhibitory peptide P60	Melanoma	Tumor immunotherapy	[123]
	SsPalm	Activated by pH change	N/A	Tumor and protozoa infection	Induce strong anti-tumor or antiprotozoal effect	[164]
	N/A	Promote gene silencing in DCs	siRNA	Tumor	Enhance tumor immunotherapy	[165]
	N/A	Direct adjuvant to draining LNs	Cyclic dinucleotides	Vaccine adjuvants	Enhance the efficacy of the adjuvant significantly	[166]
	PS	Accumulate and retain effectively in sentinel LNs	N/A	Probe for selective detection	Tumor immunotherapy	[167]
	Mannose	Increase the uptake of macrophages	N/A	HIV	Increase the absorption of in the lymphatic system	[119]
	Cholesterol	Improve transportation ability and safety	1V209 (a TLR7 agonist)	Tumor	Induce safe and durable anti-tumor immunity response	[168]
Micelle	mPEG-DSPE	Cause less tissue damage	Adriamycin	Tumor	Increase the uptake of Adriamycin	[176]
	mPEG-PLA and PLGA/mPEG-PLA	Have no toxicity to immune cells	N/A	Tumor	Act as an agonist for TLR7	[177]
	N/A	Deliver effective immunostimulatory small molecules	N/A	Tumor	Inhibit systemic inflammation and stimulate the strong immune activity	[178]
	Polyethylene glycol-b-polyaspartic acid	Have pH-triggered drug release	Epirubicin	Breast cancer	Inhibit tumor growth and axillary lymph node metastasis	[180]
	PEG-PE and PSA	Increase uptake and prolong the retention of APCs in LNs	Trp2 peptides and CpGODN	Cancer vaccines therapy	Expand antigen-specific cytotoxic T lymphocytes	[124]
	MPDA and PVP	Improve lymphatic drainage, transport and retention ability	Toll-like receptor 7 agonist imiquimod (R837)	Melanoma	Active effective DC and CD8^+^ T-cell response	[182]
	PluronicF-127	Lower risk of LN metastasis	N/A	Tumor	Increase CD8^+^ T cells in LNs and slow down tumor growth	[184]
	N/A	Change the pharmacokinetic characteristics of drugs	Toll-like receptor 7/8 agonists	N/A	Achieve effective lymphatic transport	[185]
Micelle	Pluronic and PPS	Activate complement cascades and produce danger signals	N/A	N/A	Activate DCs effectively	[186]
	PCL-PEI and PCL-PEG	Have low toxicity	Trp2 peptides and CpG oligonucleotides	N/A	Have high efficacy on DCs	[189]
	N/A	Target tumor lymphatic vessels and gather near blood vessels	LYP-1	Tumor	Have the better anti-tumor effect in vitro	[126]
	PEG-PLGA	Better distribution	LYP-1	Tumor	Achieve better anti-tumor effects	[127]
	poly (lactide-glycolide)	Prolong the residence time and activate DCs more persistently	PolyIC	Therapeutic or prophylactic vaccines	Produce a certain immune enhancement effect	[120]
Inorganic nanoparticle	Neutral polyethylene glycol polyalloy nanorods	Achieve local photothermal therapy	N/A	Tumor	Have clear inhibitory effects on tumor metastasis of LNs	[196]
	AuNP with octyl mercaptan and 11-mercaptoundecane sulfonic acid	Inhibited the growth of large tumors and prolong the survival time	TLR7 ligands	Tumor	Cause local immune activation and stimulate the response of cytotoxic T cells	[121]
	AuNP with escherichia coli membrane	Induce and regulate immune response	N/A	Antibacterial vaccine	Result in a strong antibody response	[199]
Inorganic nanoparticle	Au-SGSH	Target DNA vaccine to APCs	N/A	Tumor	Generate long-term immune response	[122]
	Lauric acid and HSA	Achieve site-specific drug delivery under the action of a localized external magnetic field	Mitoxantrone	Tumor	Have strengthen stability and linear drug release kinetics	[212]
	LHRHR and uPAR	Have small hydrodynamic diameter and high drug loading	Paclitaxel	Prostate cancer	Increase the cytotoxicity of cancer cells and reduce the concentration required for free drugs by ten times	[128]
	USPIO	Pass through lymphatic vessels faster	N/A	N/A	Gather in sentinel LNs earlier	[215]
	PEGylated DOX-Fe2+ complexes	Achieve pH-dependent drug release	Doxorubicin	Tumor	Facilitate the penetration into tumors, become less susceptible to MDR than the free drug and increase therapeutic effect	[216]
	Chitosan	Temperature-controlled drug release	Doxorubicin	N/A	Enhance therapeutic effects	[217]
	Phospholipid-PEG	Generate heat itself and benefit hyperthermia	Adriamycin	Tumor	Strengthen the effect of chemotherapy and hyperthermia in the treatment of cancer	[218]
	XL-MSN	Have high biomolecule loading	TLR9 agonist	Tumor	Enhance antigen presentation ability and increase pro-inflammatory cytokine secretion	[219]
Inorganic nanoparticle	GDMON	Change the intracellular microenvironment and ROS levels	Antigenic proteins OVA and TLR9 agonists	Tumor	Promote cytotoxic T lymphocyte proliferation and inhibit tumor growth	[220]
	RGO-PEG	Adapt to a variety of personalized new antigen peptides and transport efficiently	N/A	Nano-vaccine	Generate reactive oxygen species in DCs and induce new antigen-specific T-cell responses	[224]
	Magnetic multi-walled carbon nanotubes	Reduce lymphatic metastasis	Gemcitabine	N/A	Achieve more effective drug delivery	[225]
	Zinc phosphate and monophospholipids	Make it more difficult for tumors to escape immune surveillance	H-2kb-restricted peptides Trp2180-188 and H-2DB-restricted peptides Hgp10025-33	Subcutaneous melanoma and lung metastatic melanoma	Induce CD8^+^ T-cell response and inhibit tumor growth	[130]
	OVA modified α-alumina nanoparticles	Induce effective autophagy-dependent cross-presentation	N/A	N/A	Induce strong anti-tumor response	[228]
	Zinc-loaded ferromagnetic nano-phospholipid	Activate the immune response through TLR connection directly	PolyIC and imiquimod (R837)	Invasive B16-F10 melanoma	Induce a potent innate immune response in LNs	[229]
Hydrogel	Cholesterol pullulan nanogels	Specifically absorbed by macrophages located in the medulla	Synthetic long peptide antigens	Tumor	Present CD8^+^T cell antigen and inhibit tumor growth	[240]
	N/A	Have higher specificity and controlled release properties	Gemcitabine	Lung cancer	Reduce the toxicity and inhibit mediastinal metastasis	[242]
	Imidazoline	Improve the therapeutic benefit of local application	TLR7/8 agonist	Tumor	Induce better antibody and T-cell response and greatly reduce systemic inflammatory response	[243]
	Polyethylene glycol poly (L-valine)	Prolong the time of antigen at the injection site and increase the number of LNs	Polyinosine:polycytidine monophosphate	Melanoma	Induce cytotoxic T lymphocyte reaction and increase the number of CD8^+^ T cells in draining LNs	[131]
	Polyethylene glycol	Target multiple immune cell subsets in LNs	N/A	Cancer vaccines	Improve the ability of antigen presentation	[244]
	N/A	Affect the presence of immune cells in draining LNs	GM-CSF	Type I diabetic	Increase antigen-specific CD4^+^ T cells	[132]
Nanocapsule	Polysaccharide shells	Form a repository at the injection site	Docetaxel	Tumor	Have better biodistribution and faster access to lymphatic vessels	[246]
	N/A	Load antigens and adjuvants easily	Protein or peptide antigens	Tumor vaccines and prophylactic virus vaccines	Promote the uptake of APCs and the transport of APCs to draining LNs	[247]
Nanocapsule	N/A	Improve the oral bioavailability of insoluble drugs	Docetaxel	N/A	Increase in exposure time	[248]

## Figures and Tables

**Table 1:** Summary of the vaccine delivery systems towards lymph nodes.

Vaccine Delivery Systems	Disease	Antigen Type	Administration Routes	Delivery Efficiency	Ref.
Liposome	Malaria	Recombinant Pfs25	Intramuscularly	Enhanced several-fold	[99]
Liposome	Tumor	mRNA	Subcutaneously	Induced protein expression	[100]
Lipoprotein	Tumor	Antigens and CpG	Subcutaneously	Increased LN accumulation	[101]
Polymer	Tumor	OVA	Subcutaneously	Efficient LN accumulation	[102]
Polymer	Pneumonia	Prevnar-13	Microneedle insertion	Controlled antigen release	[103]
Polymer	Influenza	Inactivated influenza virus	Microneedle insertion	Efficient LN immune activation	[104]
Cell	Tumor	Hybrid cells	Intradermal Immunization	Immune function recovery	[105]
DNA nanodevice	Tumor	Tumor antigen peptide/CpG loop/dsRNA	Subcutaneously	Enhanced antigen-fluorescence signals in LN	[106]
Inorganic materials	Tumor	OVA	Subcutaneously	Much greater extent in LN	[107]
Peptide/protein	Chronic hepatitis B	preS1	Subcutaneously	Mainly captured by SIGNR1+ DCs and macrophages in LN	[108]
Virus	SARS-CoV-2	Prefusion stabilized spike protein	Intramuscularly/ Intranasally	Protecting upper and lower respiratory tracts	[109]

**Table 2:** Specific ligands or peptides used in nanoparticles.

Research Group	Ligand	Target	Ref.
Kaur et al.	Mannose	HIV	[119]
Jewell et al.	Poly (inosinic acid: cytidine) (PolyIC)	Therapeutic or prophylactic vaccine	[120]
Mottas et al.	TLR7 ligands	Tumor	[121]
Gulla et al.	Thiol ligands containing shikimoyl and guanidine groups	Melanoma	[122]
Liu et al.	polyethylene glycol phospholipid derivatives, anti-PD1 antibody and Treg inhibitory peptide P60	Tumor	[123]
Zeng et al.	Trp2 and TLR-9	Melanoma	[124]
Li et al.	Trp2 and CpG oligonucleotides	Tumor	[125]
Wang et al.	LYP-1	Tumor	[126]
Luo et al.	LYP-1	Tumor	[127]
Ahmed et al.	LHRHR and uPAR	Tumor	[128]
Mooney et al.	E7 peptide	Tumor	[129]
Zhuang et al.	Trp2180-188 and Hgp10025-33	Tumor	[130]
Song et al.	polypeptide hydrogel	Melanoma	[131]
Verbeke et al.	BDC peptides	Diabetes	[132]

**Table 3:** Benefits and limitations of different types of nanomaterial-based drug-delivery systems.

Type	Advantage/Benefit	Deficiency/Limitation
Liposome	Good controllability of organizational distribution Long term effect Low toxicity Multiple ways of drug administration Slow-release drug delivery Good modifiability	Difficult for industrialized production Low encapsulation efficiency of water-soluble drugs Poor stability, easy hydrolyzed, and oxidized
Micelle	Improve the water solubility of drugs Highly stable structure Low toxicity Highly functional structure	Instability in the blood circulatory system
Inorganic nanoparticle	Designed in a variety of sizes, structures and geometric shapes Good biocompatibility and stability Have a high specific surface area Different drug loading scales	Low solubility Low clearance rate in vivo Possible long-term potential toxicity Induced cytotoxicity
Hydrogel	Prevent protein denaturation Low toxicity Long term effect	Slow response rate Poor mechanical strength
Nanocapsule	Better biodistribution Better bioavailability Protect from protease and nuclease degradation	Low entrapment efficiency Low drug loading

**Table 4:** Types of therapeutic cargo loaded into exosomes.

Type	Research Group	Therapeutic Cargo	Ref.
Interfering RNAs	Munoz et al.	Cy5-anti-miR-9	[252]
	Ohno et al.	Let-7a	[253]
	Xin et al.	miR-133b	[254]
	Pan et al.	miR-122	[255]
	Kosaka et al.	miR-143	[256]
	Katakowski et al.	miR-146b	[257]
	Zhang et al.	miR-150	[258]
	Bryniarski et al.	miR-150	[259]
	Chen et al.	miR-214	[260]
	Pan et al.	shNS5b, shCD81	[255]
	Alvarez-Erviti et al.	GAPDH siRNA and BACE1 siRNA	[261]
	Wahlgren et al.	MAPK1 siRNA	[262]
	Shtam et al.	siRNA against RAD51 and RAD52	[263]
Other types of therapeutic cargo	Sun et al.	Curcumin	[264]
	Zhuang et al.	Curcumin and JSI-124	[265]
	Maguire et al.	Adeno-associated viral vector	[266]
	Mizrak et al.	Cytosine deaminase (CD) fused with uracil phosphoribosyltransferase (UPRT) and EGFP	[267]
Other types of therapeutic cargo	Hood et al.	Superparamagnetic iron oxide nanoparticles (SPION5)	[268]
	Jang et al.	Doxorubicin	[269]
	Tian et al.	Doxorubicin	[270]

**Table 5:** Studies of nanomaterials-based T-cell cancer immunotherapies.

Nanomaterials	Cargo Molecules	Disease	Ref.
Poly(beta-amino ester)-based nanomaterial	Plasmids encoding a 194-1BBz CAR and a piggyBac transposase	N/A	[275]
Liposome	IL-2–Fc fusion protein	Mouse melanoma	[276]
Liposome	TGF-β inhibitor (SB525334)	Mouse melanoma	[277]
PLGA–PEG nanomaterial	TGF-β receptor inhibitor (SD-208)	Mouse colon cancer	[278]
T-cell (Treg)-targeted hybrid nanomaterial	STAT3/STAT5 pathway inhibitor (imatinib)	Mouse melanoma	[279]
Iron nanomaterial	Anti-CD137 and anti-PD-L1	Mouse melanoma	[280]
Liposome-coated polymeric gel	Mouse IL-2 and a TGF-β inhibitor (SB505124)	Mouse melanoma	[281]
Macroporous alginate scaffolds	IL-15 superagonists, antibodies for CD3, CD28, and CD137	Mouse breast cancer, mouse ovarian cancer	[282]
Nickel–titanium alloys	Antibodies for CD3, CD28, and CD137	Mouse model of human pancreatic cancer expressing receptor tyrosine kinase-like orphan receptor (ROR1)	[283]

**Table 6:** FDA-approved nano-medicines.

Type	Drug	Date of Approval	Application	Company
Liposome	Onpattro	2018	Transthyretin-mediated amyloidosis	Alnylam Pharmaceuticals
	Vyxeos	2017	Acute myeloid leukaemia	Jazz Pharmaceuticals
	Onivyde	2015	Metastatic pancreatic cancer	Ipsen
	Marqibo	2012	Acute lymphoblastic leukaemia	Acrotech Biopharma
	Visudyne	2000	Wet age-related macular degeneration, myopia, and ocular histoplasmosis	Bausch and Lomb
	AmBisome	1997	Fungal/protozoal infections	Gilead Sciences
	DaunoXome	1996	Kaposi’s sarcoma	Galen
	Doxil	1995	Kaposi’s sarcoma, ovarian cancer, and multiple myeloma	Janssen
Polymer-based	ADYNOVATE	2015	Hemophilia	Takeda
	Plegridy	2014	Multiple sclerosis	Biogen
	Cimiza	2008	Crohn’s disease, rheumatoid arthritis, psoriatic arthritis, ankylosing spondylitis	UCB
	Abraxane	2005	Lung cancer, metastatic breast cancer, and metastatic pancreatic cancer	Celgene
	Neulasta	2002	Neutropenia, chemotherapy induced	Amgen
	Eligard	2002	Prostate cancer	Tolmar
	PegIntron	2001	Hepatitis C infection	Merck
	Copaxone	1996	Multiple sclerosis	Teva
	Oncaspar	1994	Acute lymphoblastic leukaemia	Servier Pharmaceuticals
Inorganic	Injectafer	2013	Iron-deficient anaemia	American Regent
	Feraheme	2009	Iron deficiency in chronic kidney disease	AMAG
	Venofer	2000	Iron deficiency in chronic kidney disease	American Regent
	Ferrlecit	1999	Iron deficiency in chronic kidney disease	Sanofi
	DexFerrum	1996	Iron-deficient anaemia	American Regent
	INFeD	1992	Iron-deficient anaemia	Allergan

## Data Availability

Not applicable.

## Abbreviations

AIDS	acquired immune deficiency syndrome
APCs	antigen-presenting cells
AuNPs	gold nanoparticles
CTL	cytotoxic T lymphocytes
DCs	dendritic cells
DDA	dimethyldioctadecylammonium
DDAB	dimethyldioctadecylammonium bromide
DDS	drug-delivery systems
DOX	doxorubicin
EPR	enhanced permeability and retention
GDMON	glutathione-depleted dendritic mesoporous organosilica nanoparticles
IONs	iron oxide nanoparticles
LHRHR	luteinizing hormone-releasing hormone receptor
LNs	lymph nodes
MDR	multidrug resistance
MHC x	major histocompatibility complex
mPEG-DSPE	methyl polyethylene glycol distearyl phosphatidylethanolamine
MRI	magnetic resonance imaging
MSNs	mesoporous silica nanoparticles
NK cells	natural killer cells
OND	oxyboradiene
OVA	ovalbumin
PDT	photothermal therapy
PEG	polyethylene glycol
PLA	polylactic acid
PLGA	poly (Dmuryl L-lactic acid-glycolic acid)
PolyIC	poly (inosinic acid: cytidine)
PS	1-dioleoyl-n-glycerophosphate serine
PTT	photothermal therapy
PTX	paclitaxel
PVP	polyvinylpyrrolidone
uPAR	urokinase type plasminogen activator receptor
